# Older meningioma patients: a retrospective population-based study of risk factors for morbidity and mortality after neurosurgery

**DOI:** 10.1007/s00701-022-05336-1

**Published:** 2022-08-18

**Authors:** David Löfgren, Antonios Valachis, Magnus Olivecrona

**Affiliations:** 1 Department of Oncology, Faculty of Medicine & Health, Örebro University, 70182 Örebro, SE Sweden; 2 Department of Neurosurgery, Faculty of Medicine & Health, Örebro University, 70182 Örebro, SE Sweden

**Keywords:** Meningioma, Surgical complications, Elderly, Neurosurgical complications, Perioperative complications

## Abstract

**Background:**

Meningioma is the most common primary CNS tumour. Most meningiomas are benign, and most patients are 65 years or older. Surgery is usually the primary treatment option. Most prior studies on early surgical outcomes in older patients with meningioma are small, and there is a lack of larger population-based studies to guide clinical decision-making. We aimed to explore the risks for perioperative mortality and morbidity in older patients with meningioma and to investigate changes in surgical incidence over time.

**Methods:**

In this retrospective population-based study on patients in Sweden, 65 years or older with surgery 1999–2017 for meningioma, we used data from the Swedish Brain Tumour Registry. We analysed factors contributing to perioperative mortality and morbidity and used official demographic data to calculate yearly incidence of surgical procedures for meningioma.

**Results:**

The final study cohort included 1676 patients with a 3.1% perioperative mortality and a 37.6% perioperative morbidity. In multivariate analysis, higher age showed a statistically significant association with higher perioperative mortality, whereas larger tumour size and having preoperative symptoms were associated with higher perioperative morbidity. A numerical increased rate of surgical interventions after 2012 was observed, without evidence of worsening short-term surgical outcomes.

**Conclusions:**

Higher mortality with increased age and higher morbidity risk in larger and/or symptomatic tumours imply a possible benefit from considering surgery in selected older patients with a growing meningioma before the development of tumour-related symptoms. This study further underlines the need for a standardized method of reporting and classifying complications from neurosurgery.

**Supplementary Information:**

The online version contains supplementary material available at 10.1007/s00701-022-05336-1.

## Introduction

Meningioma is the most common primary CNS tumour diagnosis, representing approximately 40% of all primary CNS tumours [19, 22]. With a median age at diagnosis of 66 years, most patients with a meningioma are 65 years or older, and there is a reported approximate 2.3:1 female predominance [19, 22]. The majority of meningiomas are benign, with a good prognosis. Ostrom et al. reports 10-year survival rates of 83.7% in benign meningiomas but with a worse prognosis with higher age and with malignant meningioma [22].

Although observation is the recommended therapeutic strategy for incidental, asymptomatic suspected meningiomas, surgery remains the primary treatment option for patients in good clinical condition and with rapidly growing or symptomatic tumours [13].

Prior studies on early surgical outcomes in older patients with meningioma have, with a few exceptions, been small, single-institution studies which are prone to selection bias, thus making the generalizability of the results questionable [1, 4, 12, 14, 24].

This warrants the need for larger, population-based studies to get results applicable to the clinical decision-making regarding older patients with meningioma.

In this study, we used real-world data from a national quality registry to investigate the pattern of surgical procedures for meningioma in older patients over time and in relation to demographic changes and explore risks for complications during the perioperative period.

## Methods

### Study design

We used data from the Swedish Brain Tumour Registry (SBTR) to perform a retrospective population- and registry-based study including all patients in Sweden who, according to the SBTR, had surgery for meningioma of any type at an age of 65 years or older between the years 1999 and 2017.

### The Swedish Brain Tumour Registry

The SBTR is a nationwide registry that collected data on patients with brain tumours from 1999 to the end of 2017 when the registry was closed. All patients in Sweden with surgery for a primary brain tumour were included. The SBTR has, historically, had a near complete coverage in three of the six Swedish geographical healthcare regions [3]. In addition, a fourth region has retrospectively completed their data to reach an almost complete coverage and is, thus, included as a fourth high-coverage region in this study [25]. These four healthcare regions cover approximately 60% of the Swedish population, covering both urban and rural areas [29].

### Study cohort

For the present study, all patients from the four SBTR high-coverage regions with a reported diagnosis of meningioma, a surgical date between 1999 and 2017 and age at surgery of 65 years or older were included. Diagnosis was determined from the reported SNOMED morphology code. All meningioma codes in the SBTR (code 953X/X) were included. Meningeal sarcomatosis (code 9539/3) was not included as it represents a primary sarcoma of the central nervous system [21]. The SNOMED coding table is available as Supplementary material (Section A).

The age of 65 years or older was chosen as this is a commonly used cut-off age for defining an older patient population [6, 12, 15, 24, 26].

### Variables

Basic patient characteristics were collected or calculated from available data in the SBTR. Age was defined as age at date of surgery. Preoperative symptoms were available as three different variables: preoperative seizures, focal deficits and symptoms of increased intracranial pressure. During the initial study period (1999–2005), only focal deficits were registered. As a result, reporting and analyses using preoperative symptoms included only patients who underwent surgery from 2006 to 2017.

WHO/ECOG performance status (WHO-PS) was available as a preoperative variable and used in this study as a surrogate marker for being frail [20]. Being frail was defined as WHO-PS 3 or 4 (corresponding to a Karnofsky performance status of 40 or lower), whereas patients with WHO-PS of 2 were categorized as intermediate and those with WHO-PS 0 or 1 as fit.

Tumour size was reported between 2006 and 2015, defined in the SBTR as the largest diameter divided into three groups: < 4 cm, 4–6 cm or > 6 cm.

Tumour site has been reported in different ways throughout the years. From 2006 and onwards, it was possible to report multifocality (yes/no) as well as laterality, bilateral, posterior cranial fossa, skull base and central location (by multiple choice). For the present study, we combined these variables to form three groups: multifocal tumour, skull base (posterior fossa, skull base or central location) and supratentorial.

We divided the tumours according to WHO tumour grade into two groups (grade 1 and grades 2–3) using the SNOMED morphology codes (coding is available as Supplementary material, Section A).

Type of surgical intervention was reported as either biopsy, resection or radical resection in 1999–2015 with the addition of near radical resection from 2016. Simpson grading (1–5) was available from 2009 [28]. We used the Simpson grade when available and “type of surgical intervention” when Simpson grade was missing. For calculations, the combined information was dichotomized into radical resection (representing Simpson grades 1–3) and partial resection or biopsy (representing Simpson grades 4–5).

SBTR variables used for this study with years of availability and details on variable characteristics are presented as Supplementary material (Section B). Further details on data entry is available as Supplementary material (Section C).

Results were divided into the two surgical periods 1999–2008 and 2009–2017 for the purpose of baseline comparisons over time.

### Outcome variables

Date of death was included in the registry from official sources at the Swedish Tax Agency (Skatteverket). Death within the first 30 days of surgery was defined as death attributed to surgery (perioperative mortality).

Perioperative complications were recorded (as yes/no) from the start of the SBTR in 1999 using three variables (local infection, local hematoma and thromboembolism) with the addition of new seizures, new or worsened focal deficits and reoperation (due to side effects) from 2006. For the purpose of the present study, a combined variable (perioperative morbidity) was created representing any type of perioperative complication. The combined variable is reported from 2006 and onwards due to the changes in variables. According to registry instructions, all complications registered occurred within the first 30 days after surgery.

### Statistics

We presented age at surgery as median and interquartile range with variance between surgical periods analysed using Mann–Whitney *U* test. Categorical data variables were summarized using descriptive statistics. When possible, *p* values were calculated using Pearson’s χ^2^ test. The analysis of incidence rates of surgical procedures per year in relation to the age-specific population used logistic regression with Performed surgery (yes/no) as the dependent variable and year of surgery as the only independent variable. Demography data were collected from official sources at Statistics Sweden (Statistiska centralbyrån) [29].

Crude risk estimates with odds ratios (OR), confidence intervals (CI) and *p* values for perioperative morbidity and perioperative mortality were calculated using univariate logistic regression.

To calculate adjusted ORs and their corresponding CI for these outcomes, we used logistic regression with the following predefined independent variables (entered simultaneously): age, sex, WHO-PS frailty groups, preoperative symptoms present, tumour site, tumour size, type of surgical intervention (dichotomized Simpson grading) and tumour type.

Due to the different uses of variables in various periods, only data from years 2009–2015 were used for univariate and multivariate analyses (with the exception of “age groups and perioperative mortality” where the entire time span was used for univariate logistic regression).

IBM SPSS Statistics for Windows, version 25.0, Armonk, NY, USA, was used for all statistical calculations. Microsoft Excel 2016 was used for initial sorting, calculating legal sex, date of birth and for calculating time from surgery to date of death.

Statistical significance level was set to *p* < 0.05, and all CIs are at the 95% confidence level.

## Results

### Study cohort

**Fig. 1:**
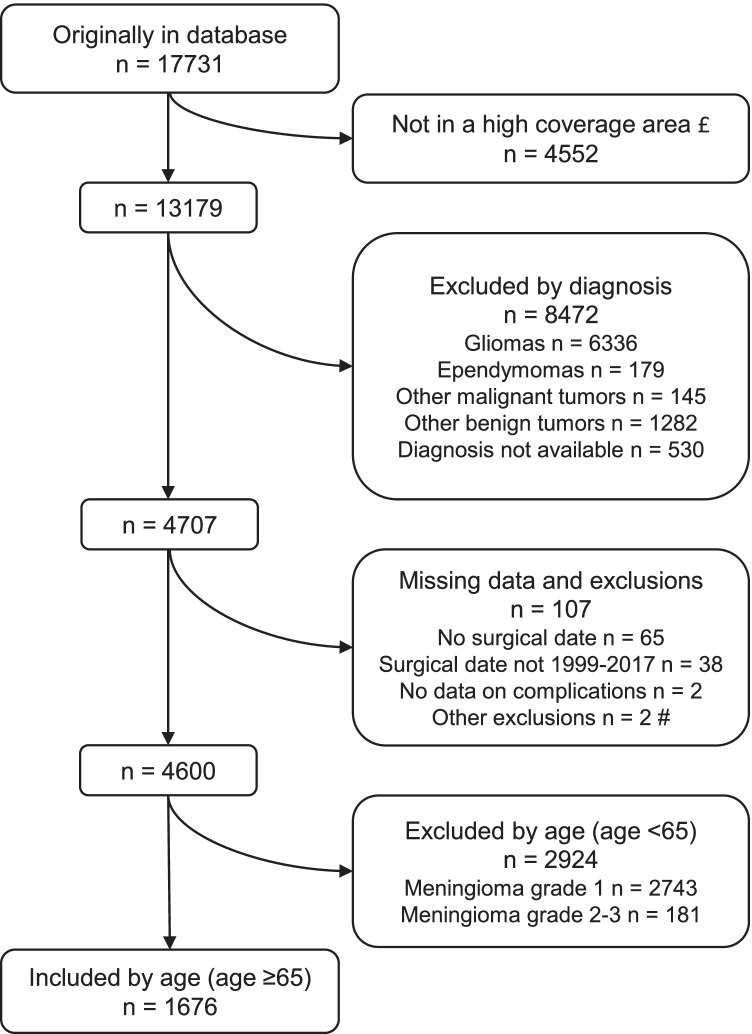
Study flowchart. Data selection and reasons for exclusion. £, as described in the Methods sections; #, date of surgery superseded by official date of death

We included 1676 patients to the final study cohort after initial sorting. Exclusion and data selection from the initial 17,731 records received from the SBTR are depicted in Fig. 1. The study population characteristics are reported in Table 1. Visual distribution of age by sex is available as Supplementary material (Section D).

**Table 1:** Baseline characteristics

			Surgical period		
Variable		Total	1999–2008	2009–2017	*p* value
Total number of patients	*N*	1676	715	961	
Age	Median (IQR)	72 (68–76)	72 (68–77)	72 (68–76)	0.291
Age groups	N (%)				
65–69		592 (35.3)	250 (35.0)	342 (35.6)	0.180
70–74		508 (30.3)	209 (29.2)	299 (31.1)	
75–79		374 (22.3)	177 (24.8)	197 (20.5)	
80 +		202 (12.1)	79 (11.0)	123 (12.8)	
Sex	Female	1137 (67.8)	499 (69.8)	638 (66.4)	0.140
	Male	539 (32.2)	216 (30.2)	323 (33.6)	
Preoperative symptoms	*N* (% of valid per variable)				
Any symptoms present		1023 (87.1) ^$^	188 (88.3) ^#^	835 (86.9)	0.588
Focal deficit		721 (66.8) ^$^	128 (63.7) ^#^	593 (67.5)	0.295
Seizures		249 (23.3) ^$^	47 (24.5) ^#^	202 (23.0)	0.662
Symptoms of intracranial pressure		443 (41.4) ^$^	66 (34.4) ^#^	377 (42.9)	0.029
WHO/ECOG performance status	*N* (% of valid)	*N* = 1644	*N* = 708	*N* = 936	
	0	630 (38.3)	332 (46.9)	298 (31.8)	< 0.001
	1	517 (31.4)	189 (26.7)	328 (35.0)	
	2	281 (17.1)	124 (17.5)	157 (16.8)	
	3	187 (11.4)	49 (6.9)	138 (14.7)	
	4	29 (1.8)	14 (2.0)	15 (1.6)	
By frailty group	Fit (0–1)	1147 (69.8)	521 (73.6)	626 (66.9)	< 0.001
	Intermediate (2)	281 (17.1)	124 (17.5)	157 (16.8)	
	Frail (3–4)	216 (13.1)	63 (8.9)	153 (16.3)	
Tumour size	*N* (% of valid)	*N* = 736 ^€^	*N* = 149 ^#^	*N* = 587 ^£^	
< 4 cm		360 (48.9) ^€^	63 (42.3) ^#^	297 (50.6) ^£^	0.192
4–6 cm		278 (37.8) ^€^	64 (43.0) ^#^	214 (36.5) ^£^	
> 6 cm		98 (13.3) ^€^	22 (14.8) ^#^	76 (12.9) ^£^	
Tumour site	*N* (% of valid)	*N* = 1535 ^$^	*N* = 577 ^#^	*N* = 958	< 0.001
Supratentorial		1148 (74.8) ^$^	442 (76.6) ^#^	706 (73.7)	
Skull base		266 (17.3) ^$^	112 (19.4) ^#^	154 (16.1)	
Multifocal		121 (7.9) ^$^	23 (4.0) ^#^	98 (10.2)	
Tumour grade	*N* (%)				
WHO grade 1		1502 (89.6)	669 (93.6)	833 (86.7)	< 0.001
WHO grade 2–3		174 (10.4)	46 (6.4)	128 (13.3)	
Type of surgical intervention	*N* (% of valid)				
Radical resection (Simpson 1–3)		1420 (84.9)	608 (85.5)	812 (84.5)	0.565
Partial res./biopsy (Simpson 4–5)		252 (15.1)	103 (14.5)	149 (15.5)	

*N*, numbers; *IQR*, interquartile range. Years: $, 2006–2017; €, 2006–2015; #, 2006–2008; £, 2009–2015

The overall female/male ratio was approximately 2:1 (68%, *n* = 1137 female, and 32%, *n* = 539 male).

Most patients (87.1%, *n* = 1023) had one or more preoperative symptom/-s. From the 1000 patients with specified preoperative symptoms 63.5% (*n* = 635) had one, 32.3% (*n* = 323) had two, and 4.2% (*n* = 42) had three.

The differences in patient and tumour characteristics between the surgical periods proved statistically significant concerning WHO-PS (including frailty groups), tumour site, tumour grade and for having preoperative symptoms of increased intracranial pressure. Notably 10.2% (*n* = 98) had a multifocal tumour in the later period, compared to 4.0% (*n* = 23) in the earlier.

### Perioperative mortality

The overall 30-day mortality was 3.1% (*n* = 52) as depicted in Table 2, with no statistically significant difference between the surgical periods.

**Table 2:** Perioperative outcomes

			Surgical period		
Variable		Total	1999–2008	2009–2017	*p* value
Perioperative mortality	*N* (%)	52 (3.1)	24 (3.4)	28 (2.9)	0.605
Perioperative morbidity	*N* (% of valid per variable)				
Any complication		441 (37.6) ^$^	76 (35.7) ^#^	365 (38.0)	0.530
Local infection		111 (9.5) ^$^	17 (8.0) ^#^	94 (9.8)	0.417
Local hematoma		193 (16.4) ^$^	25 (11.7) ^#^	168 (17.5)	0.041
Thromboembolism		51 (4.4) ^$^	7 (3.3) ^#^	44 (4.6)	0.416
New seizures		96 (8.2) ^$^	13 (6.3) ^#^	83 (8.6)	0.278
New focal deficit		241 (20.7) ^$^	44 (21.6) ^#^	197 (20.5)	0.732
Reoperation		70 (6.0) ^$^	14 (6.8) ^#^	56 (5.8)	0.583
Cause for reoperation	*N* (% of reoperations)				
Reoperation and local infection		14 (20.0) ^$^	4 (17.9) ^#^	10 (17.9)	0.144
Reoperation and local hematoma		32 (45.7) ^$^	3 (21.4) ^#^	29 (51.8)	
Reoperation, local infection and hematoma		14 (20.0) ^$^	3 (21.4) ^#^	11 (19.6)	

*N*, numbers; *IQR*, interquartile range. Years: $, 2006–2017; #, 2006–2008

Among patients who died within 30 days of surgery during the years with consistent reporting of complications (2006–2017), perioperative complications were simultaneously registered in 66.7% (*n* = 22). Worsened neurological function was the most common simultaneous complication with 81.8% (*n* = 18), followed by local hematoma with 59.1% (*n* = 13), reoperation due to side effects with 31.8% (*n* = 7), perioperative local infection with 27.3% (*n* = 6), new or worsened seizures with 27.3% (*n* = 6) and thromboembolism with 9.1% (*n* = 2).

Unadjusted and adjusted ORs for perioperative mortality are summarized in Table 3. While being frail (determined by WHO-PS), having a larger than 6 cm tumour and higher age showed statistically significant results in the univariate logistic regression, only higher age remained with a statistically significant influence to the outcome perioperative mortality in multivariate analysis. Hosmer and Lemeshow test for goodness of fit shows support for our adjusted model (*p* = 0.458).

**Table 3:** Perioperative mortality

Variable	Crude OR (CI)	*p* value	Adjusted OR (CI)	*p* value
Age at surgery	1.134 (1.048–1.226)	0.002	1.135 (1.026–1.255)	0.014
Sex (f/m)	1.086 (0.422–2.793)	0.865	1.528 (0.487–4.790)	0.467
WHO performance status		0.003 ^#^		0.318 ^#^
0–1 vs 2	3.172 (0.839–12.001)	0.089	1.649 (0.360–7.554)	0.520
0–1 vs 3–4	7.146 (2.291–22.289)	< 0.001	2.843 (0.734–11.016)	0.131
Preoperative symptoms (no vs yes)				
Symptoms present	2.753 (0.363–20.869)	0.327	1.076 (0.122–9.493)	0.948
Focal deficit	0.942 (0.353–2.515)	0.905		
Seizures	1.895 (0.733–4.898)	0.187		
Symptoms of intracranial pressure	0.764 (0.298–2.116)	0.644		
Tumour site		0.445 ^#^		0.079 ^#^
Supratentorial vs skull base	1.974 (0.682–5.717)	0.210	3.912 (1.039–14.728)	0.044
Supratentorial vs multifocal	1.437 (0.314–6.573)	0.640	4.098 (0.721–23.291)	0.112
Tumour size		0.017 ^#^		0.298 ^#^
< 4 cm vs 4–6 cm	2.113 (0.589–7.581)	0.251	1.464 (0.354–6.057)	0.599
< 4 cm vs > 6 cm	6.279 (1.725–22.848)	0.005	3.191 (0.700–14.542)	0.134
Type of surgery				
Radical vs non-radical/biopsy	2.649 (0.985–7.127)	0.054	3.185 (0.971–10.446)	0.056
Tumour type				
WHO grade 1 vs 2–3	2.306 (0.812–6.551)	0.117	1.280 (0.308–5.326)	0.734

Odds ratio and 95% confidence interval for perioperative morbidity; #, variable *p* value

Table 4 shows the association of age (in 5-year groups) with perioperative mortality, showing statistically significant higher risk of perioperative mortality in the two oldest age groups (Pearson’s χ^2^ test: *p* < 0.001).

**Table 4:** Age groups and perioperative mortality

	Perioperative mortality			
Age at surgery	No, *n* (%)	Yes, *n* (%)	Crude OR (CI)	*p* value
65–69	585 (98.8)	7 (1.2)		
70–74	496 (97.6)	12 (2.4)	2.022 (0.790–5.175)	0.142
75–79	359 (96.0)	15 (4.0)	3.492 (1.410–8.646)	0.007
> 80	184 (91.1)	18 (8.9)	8.175 (3.362–19.881)	< 0.001

Numbers and percentage for each age group. Unadjusted OR and 95% CI for perioperative mortality from logistic regression with age 65–69 as index. Variable *p* value < 0.001

### Perioperative morbidity

Perioperative morbidity is presented in Table 2. In total, 37.6% (*n* = 441) suffered from perioperative morbidity of any kind. There were no statistically significant differences in overall perioperative morbidity between the surgical periods, nor regarding any of the available types of complications, except for local hematoma. Complications per patient showed no statistically significant changes between the surgical periods (*p* = 0.811), and in total 19.7% (*n* = 231) had one, 10.2% (*n* = 119) had two, and 7.6% (*n* = 89) had three or more listed complications.

The summary of OR for perioperative morbidity is depicted in Table 5. Univariate analysis showed being frail (determined by WHO-PS), having preoperative symptoms, having a larger tumour or the tumour being WHO grades 2–3, as statistically significantly correlated to a higher risk of suffering from perioperative morbidity. In the multivariate analysis, however, only larger tumour size and having preoperative symptoms made an independent statistically significant contribution to the outcome. Hosmer and Lemeshow test for goodness of fit indicates support for the adjusted model (*p* = 0.277).

**Table 5:** Perioperative morbidity

Variable	Crude OR (CI)	*p* value	Adjusted OR (CI)	*p* value
Age at surgery	1.011 (0.983–1.039)	0.458	0.998 (0.965–1.033)	0.916
Sex (f/m)	1.112 (0.812–1.522)	0.509	1.025 (0.700–1.503)	0.898
WHO performance status		0.046 ^#^		0.136 ^#^
0–1 vs 2	0.884 (0.578–1.351)	0.569	0.766 (0.465–1.261)	0.295
0–1 vs 3–4	1.615 (1.064–2.453)	0.024	1.413 (0.859–2.324)	0.173
Preoperative symptoms (no vs yes)				
Symptoms present	1.823 (1.123–2.960)	0.015	2.030 (1.128–3.654)	0.018
Focal deficit	1.522 (1.074–2.157)	0.018		
Seizures	1.445 (1.008–2.072)	0.045		
Symptoms of intracranial pressure	0.928 (0.671–1.283)	0.650		
Tumour site		0.184 ^#^		0.254 ^#^
Supratentorial vs skull base	0.829 (0.543–1.266)	0.385	0.805 (0.483–1.340)	0.404
Supratentorial vs multifocal	1.488 (0.881–2.514)	0.137	1.505 (0.810–2.794)	0.196
Tumour size		< 0.001 ^#^		0.006 ^#^
< 4 cm vs 4–6 cm	1.747 (1.207–2.529)	0.003	1.647 (1.100–2.466)	0.015
< 4 cm vs > 6 cm	2.586 (1.545–4.328)	< 0.001	2.274 (1.294–3.997)	0.004
Type of surgery				
Radical vs non-radical/biopsy	1.204 (0.797–1.820)	0.378	1.350 (0.832–2.191)	0.224
Tumour type				
WHO grade 1 vs 2–3	1.725 (1.126–2.643)	0.012	1.447 (0.876–2.391)	0.149

Odds ratio and 95% confidence interval for perioperative morbidity; #, variable *p* value

### Incidence rates of surgical procedures over time

**Fig. 2:**
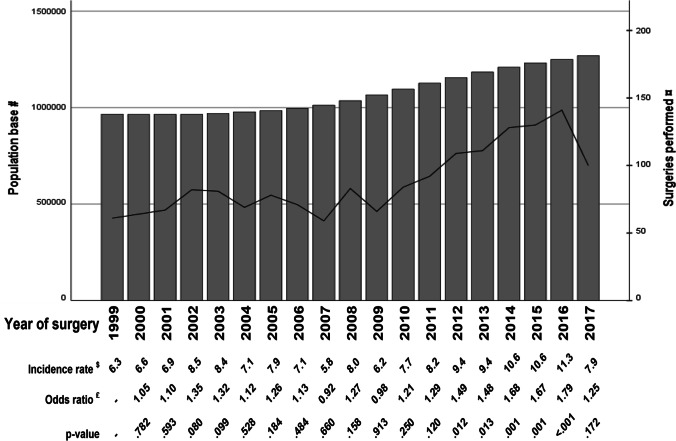
Population base and number of surgeries by year of surgery. *N* of persons age 65 years or older living in the studied healthcare regions and number of surgeries performed, incidence rate, OR for having surgery and corresponding *p* value by year of surgery. #, *N* of persons age 65 years or older (bars); ¤, *N* of surgeries performed (line); $, incidence rate of surgery per 100,000 for each year; £, OR for having surgery from univariate logistic regression with 1999 as index year. *p* value for the regression < 0.001

The comparison of number of surgeries performed each year and the population (older than 65 years) of each year is reported in Fig. 2. The overall logistic regression of number of surgeries performed on this age group over time proved statistically significant (*p* < 0.001). The increased incidence of years 2012 to 2016 was statistically significantly different from the index year 1999.

### Discussion

In this retrospective population-based registry study on patients in Sweden, 65 years or older, we showed that higher age is a major prognostic factor for perioperative mortality after meningioma surgery. In addition, larger tumour size and having preoperative symptoms were associated with increased risk for perioperative complications. These findings of risk factors for perioperative mortality and perioperative morbidity imply that it could be beneficial to consider surgery in older patients with a growing meningioma before the development of tumour-related symptoms.

Our evaluation of incidence rates of surgical procedures over time showed a relatively stable curve until 2012, with a trend towards an increased rate of surgical interventions afterwards, without evidence of worsening short-term surgical outcomes.

### Perioperative mortality

We observed a higher perioperative mortality by age which is in accordance to prior studies [1, 4, 14, 30]. The perioperative mortality rates in prior smaller cohorts which are prone to selection bias vary greatly, from 0% in some recent cohorts[17, 23] to more than 20% in some historical cohorts [10, 18]. However, our perioperative mortality rates and the finding of higher perioperative mortality with increasing age correspond well with other large cohorts as the studies by Grossman et al. (3.2% inpatient mortality, 65 years and older), Bateman et al. (4% inpatient mortality, 70 years and older) and Albert et al. (1.4% 30-day mortality in age 61–70; 2.4% in age 71–80; 6.9% in age > 80) [1, 4, 14]. In comparison to cohorts with younger patient populations, Corell et al., with data from the SBTR but with all ages included, showed a 1.5% 30-day mortality, and the younger cohorts in the study by Albert et al. had 0.7% 30-day mortality in ages 18–60, clearly demonstrating the increased perioperative mortality by age [1, 9]. High age being an independent risk factor for this outcome in multivariate analysis has been previously shown in several studies [4, 14, 30].

### Perioperative morbidity

When looking at perioperative morbidity or perioperative complications, the published studies vary greatly in terms of the occurrence of complications, as summarized in two literature reviews [12, 24]. Eksi et al. calculated the mean occurrence of postsurgical complications to 37.1% with data from 23 studies on older patients with meningioma which is in accordance to our findings with 37.6% overall perioperative morbidity [12]. Poon et al. chose not to meta-analyse complication data because of the wide range of reported complication rates (2.7–29.8% in five studies) and the heterogeneity of eligible studies [24].

Regarding cohort studies investigating the perioperative morbidity after surgery for meningioma in older patients, Bateman et al. showed 53.2% adverse outcomes in patients aged 70 years or older [4], and Grossman et al. had a total complication rate of 17.5% in patients 65 years or older [14]. These differences in outcomes highlight the need for an internationally agreed convention for defining and grading complications after meningioma surgery to enable benchmarking and valuable comparisons among different cohorts. The proposed scoring system by Clavien and Dindo is one such possibility [7, 16].

### Incidence rates of surgical procedures over time

Our evaluation of incidence of surgical interventions showed a relatively stable curve over time, with the later years 2012–2016 deviating from the expected increase caused by the increase of the age-specific population. This observation along with the absence of an increased perioperative mortality or increased perioperative morbidity over time could indicate that minor changes in indications for meningioma surgery might have occurred or that an increased overall meningioma incidence might be the reason for the transient increase in surgical procedures.

Nilsson et al. reported no evidence for increased incidence of meningioma in Sweden during partially overlapping years but rather a slight decrease in the older patient group, perhaps owing to a higher chance of early incidental discovery [19]. Speculatively, this could indicate that our slightly increased incidence of surgery might have an underlying larger increase in true incidence of performed surgery, considering the potential decrease in the incidence of meningioma in older patients.

In our cohort, there were no statistically significant differences over time regarding major preoperative symptoms, but multifocal tumours and frail patients were more common in the later surgical period. These findings imply that there might be an underlying drift in the Swedish Neurosurgical Society towards suggesting surgery for patients older than 65 years of age, with more complex meningiomas than earlier. If this is the case, this shift regarding the indication for meningioma surgery has been managed without the cost of worse short-term outcomes.

### Strengths and limitations

The major strengths of this study were the population coverage and the large number of included patients. There are few population-based multicentre studies focusing on the perioperative outcome of older meningioma patients. With 1676 patients aged 65 and above, this is one of very few studies presenting results from more than 1000 patients [1, 4, 12, 14, 24]. The large number of patients has allowed us to perform regression analyses making our findings more robust compared to smaller studies. In addition, the access to official demographic data for the covered parts of the country has made it possible to evaluate the changes in surgical incidence over time in relation to the increase of the age-specific population. The SBTR recorded patients that had undergone surgery, meaning that we do not have data to compare these changes with changes in the total numbers of patients with meningioma.

Our study has several limitations that deserve discussion. First, the outcome variables regarding perioperative morbidity were recorded without grading of severity or information on permanence. In addition, the time where each complication occurred during the perioperative period could not be determined nor the outcome of each complication. Of note, all the variables on complications were retrospectively derived from hospital records, a method that has known issues in comparison with patient reported outcome measures [11]. In the same manner, the preoperative symptom variables are dichotomous and have no grading of severity of symptoms. We can, however, assume that the symptoms must have had a high degree of impact on the patients to be recorded. Furthermore, we lack potentially relevant information (e.g. concurrent medication, comorbidities or peritumoural oedema) to assess commonly used meningioma grading scores and to provide a more comprehensive frailty assessment other than WHO-PS [2, 5, 8, 27].

An additional limitation is the differences in recorded variables throughout the years that the SBTR covers. As an example, even though records of type of surgery were available for the entire timeframe of the registry (divided in 3 or 4 levels), Simpson grading was only available from 2009. These changes in variables, including the late use of Simpson grading, limit the available years of consistent reporting and thus the number of patients available for multivariate analysis.

## Conclusion

Despite the caveats, this study presented results on the short-term outcome after surgery from one of the largest cohorts of older patients with meningioma. Higher age seems to be an independent risk factor in dying within 30 days of meningioma surgery, whereas tumour size and having preoperative symptoms from meningioma were associated with higher risk for perioperative complications. The latter findings imply that it could be beneficial to consider surgery in older patients with a growing meningioma before the development of tumour-related symptoms.

This study further underlines the need for a more standardized method of reporting and classifying complications from neurosurgery.

## Supplementary information

Below is the link to the electronic supplementary material.

Supplementary file1 (DOCX 139 KB)

## Data Availability

The datasets generated during and/or analysed during the current study are not publicly available due to their sensitive personal nature but are available from the corresponding author on reasonable request.

## Abbreviations

CI: Confidence intervals
ECOG: Eastern Cooperative Oncology Group
OR: Odds ratios
SBTR: Swedish Brain Tumour Registry
SNOMED: Systematized Nomenclature of Medicine
WHO: World Health Organization
WHO-PS: WHO/ECOG performance status
