# Supplementary material for: Older meningioma patients: a retrospective population-based study of risk factors for morbidity and mortality after neurosurgery
Source: Acta Neurochir (Wien). 2022 Aug 18;164(11):2987–97. doi: 10.1007/s00701-022-05336-1 (PMC9613739; doi:10.1007/s00701-022-05336-1)

# Supplementary Material

Older meningioma patients – a retrospective population based study of risk factors for morbidity and mortality after neurosurgery.

Section A

| SNOMED coding table, all available codes in SBTR | |  |  |
| --- | --- | --- | --- |
|  |  |  |  |
| **M-code** | **Morphology term, commonly used** | **Diagnosis group in study** | **N in SBTR** |
| 33400 | Cyst | Other benign tumours | 21 |
| 71620 | Colloid cyst | Other benign tumours | 8 |
| 72900 | Cholesteatoma | Other benign tumours | 5 |
| 80000 | Tumour, benign | Other benign tumours | 3 |
| 80001 | Tumour, unknown malignancy | Other benign tumours | 13 |
| 80003 | Tumour, malignant NOS | Other malignant tumours | 6 |
| 80203 | Carcinoma, undifferentiated, NOS | Other malignant tumours | 1 |
| 80213 | Carcinoma, anaplastic, NOS | Other malignant tumours | 1 |
| 81403 | Adenocarcinoma, NOS | Other malignant tumours | 2 |
| 82463 | Neuroendocrine carcinoma, NOS | Other malignant tumours | 2 |
| 88500 | Lipoma, NOS | Other benign tumours | 1 |
| 90643 | Germinoma | Other malignant tumours | 21 |
| 90800 | Teratoma, benign | Other benign tumours | 3 |
| 90803 | Teratoma, malignant | Other malignant tumours | 2 |
| 90840 | Dermoid cyst, NOS | Other benign tumours | 25 |
| 91200 | Hemangioma | Other benign tumours | 56 |
| 91501 | Hemangiopericytoma, unknown malignancy | Other benign tumours | 8 |
| 91503 | Hemangiopericytoma, malignant | Other malignant tumours | 11 |
| 91611 | Hemangioblastoma | Other benign tumours | 215 |
| 93501 | Craniopharyngioma | Other benign tumours | 102 |
| 93601 | Pinealoma | Other benign tumours | 11 |
| 93623 | Pineoblastoma | Other malignant tumours | 9 |
| 93630 | Melanotic neuroectodermal tumour | Other malignant tumours | 2 |
| 93801 | Glioma of uncertain malignant potential | Gliomas | 4 |
| 93803 | Glioma, malignant | Gliomas | 361 |
| 93813 | Gliomatosis cerebri | Gliomas | 5 |
| 93823 | Oligoastrocytoma gr II/III | Gliomas | 315 |
| 93831 | Subependymoma | Ependymomas | 59 |
| 93841 | Subependymal giant cell astrocytoma | Gliomas | 11 |
| 93900 | Choroid plexus papilloma, NOS | Other benign tumours | 25 |
| 93901 | Atypical choroid plexus papilloma | Other malignant tumours | 3 |
| 93903 | Choroid plexus carcinoma | Other malignant tumours | 1 |
| 93913 | Ependymoma, NOS | Ependymomas | 132 |
| 93923 | Ependymoma, anaplastic | Ependymomas | 26 |
| 93933 | Papillary ependymoma | Ependymomas | 1 |
| 93941 | Myxopapillary ependymoma | Ependymomas | 1 |
| 94001 | Diffuse astrocytoma, of uncertain malignant potential | Gliomas | 2 |
| 94003 | Diffuse astrocytoma, low grade | Gliomas | 690 |
| 94013 | Astrocytoma, anaplastic | Gliomas | 709 |
| 94103 | Protoplasmic astrocytoma | Gliomas | 1 |
| 94113 | Gemistocytic astrocytoma | Gliomas | 56 |
| 94121 | Desmoplastic infantile astrocytoma | Gliomas | 1 |
| 94130 | Dysembryoplastic neuroepithelial tumour | Other benign tumours | 21 |
| 94203 | Fibrillary astrocytoma | Gliomas | 60 |
| 94211 | Pilocytic astrocytoma | Gliomas | 107 |
| 94213 | Pilocytic astrocytoma (94211 in ICD-O-3 | Gliomas | 60 |
| 94223 | Spongioblastoma, NOS (94211 in ICD-O-3) | Gliomas | 1 |
| 94243 | Pleomorphic xanthoastrocytoma | Gliomas | 16 |
| 94253 | Pilomyxoid astrocytoma | Gliomas | 2 |
| 94401 | Does not exist in ICD-O-2/3, treated as 94403 | Gliomas | 1 |
| 94403 | Glioblastoma, NOS | Gliomas | 5055 |
| 94413 | Giant cell glioblastoma | Gliomas | 78 |
| 94423 | Gliosarcoma | Gliomas | 122 |
| 94503 | Oligodendroglioma, NOS | Gliomas | 456 |
| 94513 | Oligodendroglioma, anaplastic | Gliomas | 308 |
| 94603 | Oligodendroblastoma | Gliomas | 5 |
| 94703 | Medulloblastoma, NOS | Other malignant tumours | 45 |
| 94713 | Desmoplastic nodular medulloblastoma | Other malignant tumours | 7 |
| 94733 | Primitive neuroectodermal tumour, NOS | Other malignant tumours | 67 |
| 94743 | Large cell medulloblastoma | Other malignant tumours | 3 |
| 94900 | Ganglioneuroma | Other benign tumours | 4 |
| 94903 | Ganglioneuroblastoma | Other malignant tumours | 1 |
| 94920 | Gangliocytoma | Other benign tumours | 5 |
| 94930 | Dysplastic gangliocytoma of cerebellum | Other benign tumours | 3 |
| 95003 | Neuroblastoma, NOS | Other malignant tumours | 7 |
| 95013 | Medulloepithelioma, NOS | Other malignant tumours | 1 |
| 95030 | Does not exist in ICD-O-2/3, treated as 94130 | Other benign tumours | 8 |
| 95033 | Neuroepithelioma, NOS | Other malignant tumours | 3 |
| 95050 | Ganglioglioma, benign | Gliomas | 1 |
| 95051 | Ganglioglioma, NOS | Gliomas | 112 |
| 95053 | Ganglioglioma, anaplastic | Gliomas | 8 |
| 95060 | Central neurocytoma, benign | Other benign tumours | 3 |
| 95061 | Central neurocytoma | Other benign tumours | 26 |
| 95083 | Atypical teratoid/rhabdoid tumour | Other malignant tumours | 1 |
| 95223 | Olfactory neuroblastoma | Other malignant tumours | 26 |
| 95233 | Olfactory neuroepithelioma | Other malignant tumours | 1 |
| 95300 | Meningioma, NOS | Meningioma, grade 1 | 4543 |
| 95301 | Meningiomatosis, NOS | Meningioma, grade 1 | 30 |
| 95303 | Meningioma, malignant | Meningioma, grade 2-3 | 73 |
| 95310 | Meningothelial meningioma | Meningioma, grade 1 | 526 |
| 95320 | Fibrous meningioma | Meningioma, grade 1 | 207 |
| 95330 | Psammomatous meningioma | Meningioma, grade 1 | 63 |
| 95340 | Angiomatous meningioma | Meningioma, grade 1 | 74 |
| 95370 | Transitional meningioma | Meningioma, grade 1 | 276 |
| 95381 | Clear cell meningioma | Meningioma, grade 2-3 | 41 |
| 95383 | Papillary meningioma | Meningioma, grade 2-3 | 8 |
| 95391 | Atypical meningioma | Meningioma, grade 2-3 | 450 |
| 95393 | Meningeal sarcomatosis | Other malignant tumours | 3 |
| 95400 | Neurofibroma, NOS | Other benign tumours | 5 |
| 95403 | Malignant peripheral nerve sheath tumour | Other malignant tumours | 3 |
| 95500 | Plexiform neurofibroma | Other benign tumours | 1 |
| 95600 | Neurilemoma, NOS | Other benign tumours | 1177 |
| 95603 | Neurilemoma, malignant | Other malignant tumours | 1 |
|  |  |  |  |
|  |  | Sum - SNOMED code | 17036 |
| 99 |  | Sum - no info | 618 |
| Alter |  | Sum - "Alternative missing" | 77 |
|  |  |  |  |
|  |  | Sum - SNOMED +missing | 17731 |

Section B

| **Study Variables** |  |  |
| --- | --- | --- |
| **Variable name** | **Years available** | **Definition/explanation** |
| Personal Identity Number | 1999-2017 | Containing date of birth and sex |
| Date of surgery | 1999-2017 |  |
| Date of death | 1999-2017 | Automatically imported to registry |
| WHO/ECOG Performance status | 1999-2017 | Preoperative performance status (0-4) |
| **Preoperative symptoms** |  |  |
| Focal neurological symptoms | 1999-2017 | Preoperative symptoms caused by tumour |
| No signs of symptoms | 2006-2017 | Parent variable indicating no preoperative symptoms |
| Epilepsy | 2006-2017 | Preoperative seizures caused by tumour |
| Symptoms of intracranial pressure | 2006-2017 | Preoperative symptoms caused by tumour |
| **Tumour site** |  |  |
| Multifocal tumour | 1999-2017 | Multifocal growth pattern |
| Bilateral | 2006-2017 | Bilateral tumour growth |
| Right side | 1999-2017 | Growth in right hemisphere |
| Left side | 1999-2017 | Growth in left hemisphere |
| Frontal lobe | 1999-2005 |  |
| Temporal lobe | 1999-2005 |  |
| Parietal lobe | 1999-2005 |  |
| Occipital lobe | 1999-2005 |  |
| Cerebellum | 1999-2005 |  |
| Cerebellopontine Angle | 1999-2005 |  |
| Posterior cranial fossa | 2006-2017 |  |
| Base of skull | 1999-2017 |  |
| Brain stem | 1999-2005 |  |
| Central location | 1999-2017 | Thalamus, basal ganglia etc. |
| **Tumour size** | 2006-2015 | Size of tumour by largest diameter (<4cm ; 4-6cm ; >6cm) |
| **Type of surgical intervention** |  |  |
| Biopsy | 1999-2017 | Diagnostic biopsy only |
| Resection | 1999-2017 | Extensive surgery, not radical |
| Radical resection | 1999-2017 | Extensive surgery, gross total resection |
| Near radical resection | 2016-2017 | Extensive surgery, small residual tumour |
| **Simpson grading** | 2009-2017 | 1-5 |
| **Postoperative complications** |  |  |
| Local infection | 1999-2017 | Postoperative local infection |
| Local hematoma | 1999-2017 | Postoperative intracranial bleeding |
| Thromboembolism | 1999-2017 | Postoperative thromboembolism, any kind |
| Postoperative complications | 2009-2010 | Parent variable, any type of complication |
| New seizures | 2006-2017 | New or worsened seizures |
| New focal deficit | 2006-2017 | New or worsened focal neurological deficits |
| Reoperation | 2006-2017 | Reoperation due to postoperative complications |
| **SNOMED tumour code** | 1999-2017 | Systematized Nomenclature of Medicine, morphology |
| Study variables from the SBTR, with variable explanation and years available on report forms. | | |

Section C

Data entry and questionnaires

User handbooks and latest active report forms as well as control documents for the Swedish Brain Tumour Registry are available online (in Swedish only) at: <https://cancercentrum.se/samverkan/cancerdiagnoser/hjarna-ryggmarg-och-hypofys/hjarna-och-ryggmarg/kvalitetsregister/dokument/>

First registration and “follow up” registration (with pathology report and perioperative complications etc) was usually performed at the neurosurgical clinic where surgery was performed. Data on time laps from surgery to actual registration is not available.
Subsequent registration of postoperative treatment (e.g. radiation therapy) was performed by the respective oncology clinic.

Report form example (excerpt from registration form used from 2006-2009). The report forms were available in paper as well as online registration.


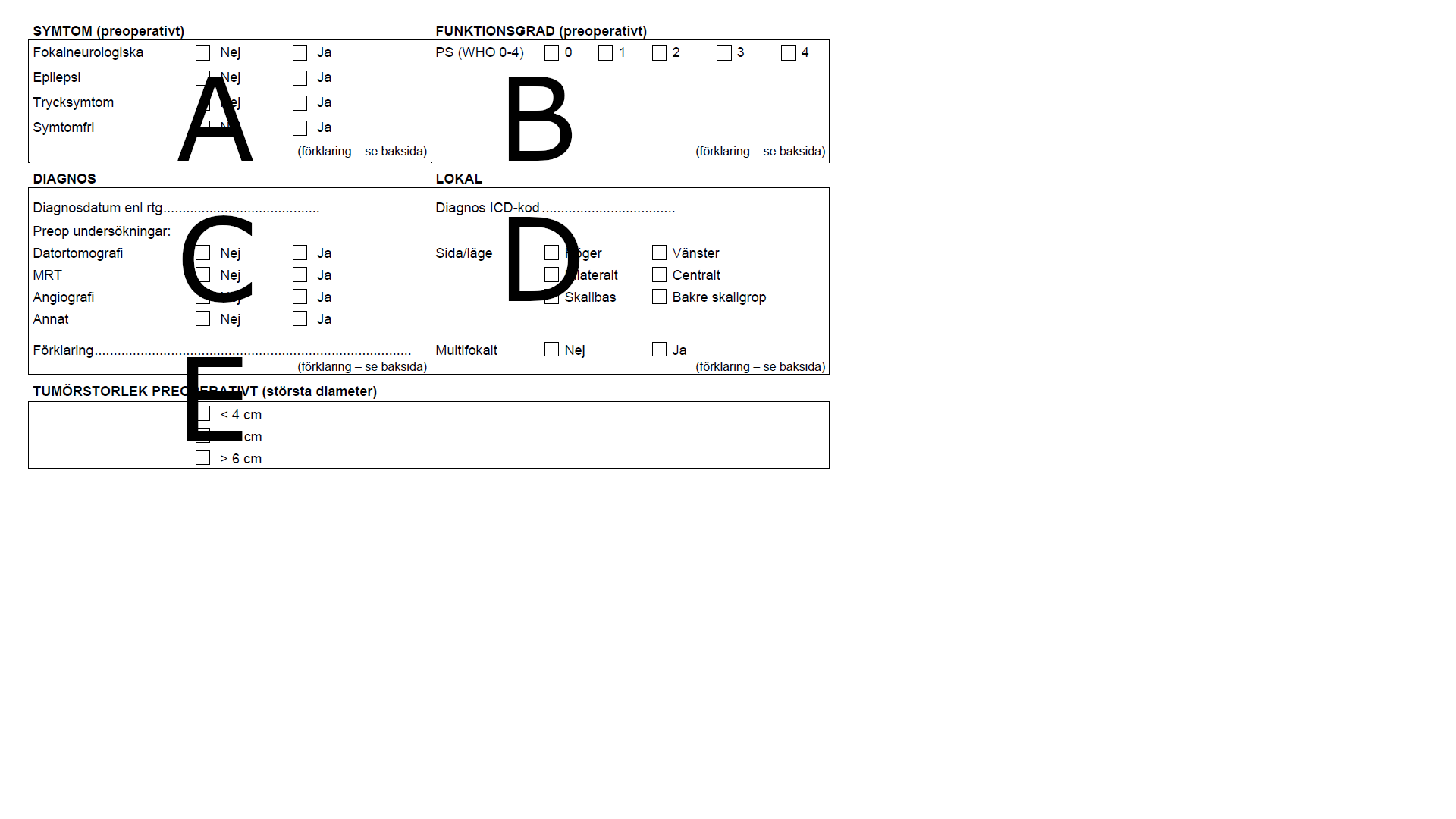


A – preoperative symptoms
Fokalneurologi (focal neurological deficits) (fokalneurologi), Epilepsi (epileptic seizures), Trycksymtom (symptoms of increased intracranial pressure) and Symtomfri (no symtoms). Available as Ja (yes) or Nej (No).

B – preoperative performance status

C – diagnosis from preoperative radiology (not used in this study)

D – Tumour site/location
Höger (right side), Vänster (left side), Bilateralt (bilateral), Centralt (central), Skallbas (skull base) and Bakre skallgrop (posterior cranial fossa) were all available in a similar fashion from 2006 and onwards. Multifokalt (multifocality) with a Nej (no) or Ja (yes) option was available from 1999 and onwards.

E – Tumour size
Defined in the Swedish brain tumour registry as the largest diameter.

Section D


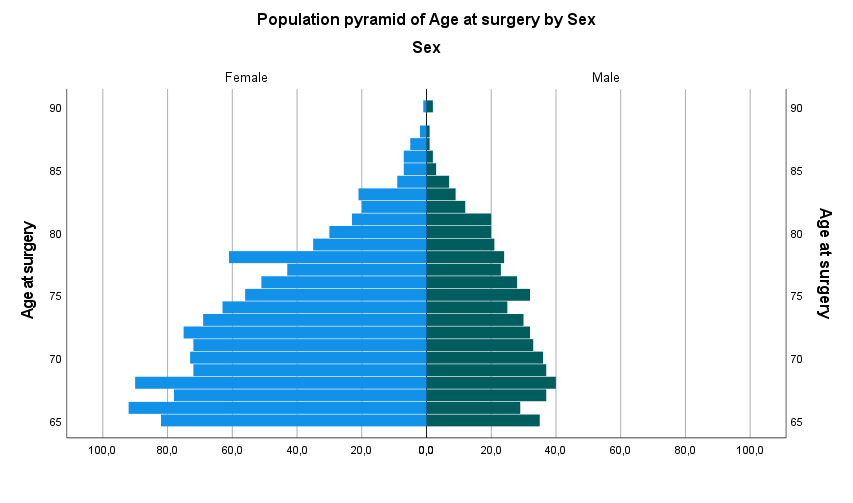

Supplement: Supplementary file 1 — Supplementary file1 (DOCX 139 KB) [file 701_2022_5336_MOESM1_ESM.docx]
